# Clinical and molecular variations in Burkitt lymphoma

**DOI:** 10.1016/j.tranon.2025.102611

**Published:** 2025-11-25

**Authors:** Eoghan O’Connor, Patricia Scanlan, Owen Patrick Smith, Melinda Halasz

**Affiliations:** a Systems Biology Ireland, UCD School of Medicine, University College Dublin, Ireland; b Muhimbili National Hospital and Tumaini la Maisha Children's Cancer Charity, Dar es Salaam, Tanzania; c National Children’s Cancer Service, Children's Health Ireland at Crumlin, Ireland; d School of Medicine, Faculty of Health Sciences, Trinity College Dublin, Ireland

**Keywords:** Lymphoma, EBV, Burkitt, Malaria, MYC

## Abstract

Burkitt lymphoma (BL) is an aggressive B-cell non-Hodgkin lymphoma, historically classified into three subtypes; endemic, sporadic and immunodeficiency associated BL. Accumulating evidence suggests that Epstein-Barr virus (EBV)-positive and EBV-negative BL represent biologically distinct entities. In this review, we aim to compare the clinicopathological and molecular differences in BL in the context of EBV status and chronic malaria infection.

From our review, clinical features of BL vary by both EBV status and geographical region, reflecting underlying epidemiological differences. The cell of origin may also differ between EBV-positive and EBV-negative cases. At a molecular level, differences based on EBV status include variations in the immunoglobulin (*IG*)::*MYC* translocation breakpoints, mutations in the inhibitor of DNA binding 3 (*ID3*)/ transcription factor 3 (*TCF3*)/ cyclin D3 (*CCND3*) signalling axis, the anti-apoptotic effects of EBV latency gene products, differences in the alternative reading frame (ARF)/ mouse double minute 2 (MDM2)/p53 and ataxia-telangiectasia mutated (ATM)/ ATM and RAD3-related (ATR) pathways, and deregulation of B-cell leukemia/lymphoma 2 (BCL-2) family proteins. We further discuss the theory that aberrant activation-induced cytidine deaminase (AID) expression, in the setting of EBV infection and chronic malaria exposure, is the most likely aetiology of endemic BL.

This review provides a comprehensive summary of key molecular differences between EBV-positive and EBV-negative BL, that may guide the development of future targeted therapeutic strategies.

## Highlights

• EBV-positive and EBV-negative Burkitt lymphoma are discrete biologic groups based on molecular features regardless of epidemiologic context and geographic location.
• The presence of apoptotic gene mutations in Burkitt lymphoma can differ significantly based on EBV status.
• EBV-negative Burkitt lymphoma is more dependent on *ID3/TCF3/CCND3* axis deregulation compared to EBV-positive Burkitt lymphoma.
• EBV-negative Burkitt lymphoma relies on oncogenic mutations in the *ARF-MDM2-TP53* apoptotic pathway, while EBV-positive Burkitt lymphoma gains anti-apoptotic activity as a direct effect of EBV on apoptotic pathways.
• Co-infection of chronic malaria with early EBV infection leads to aberrant AID expression.

## Graphical abstract

Created in BioRender. Halasz, M. (2025) https://BioRender.com/g9ln8jq.

**Figure:**
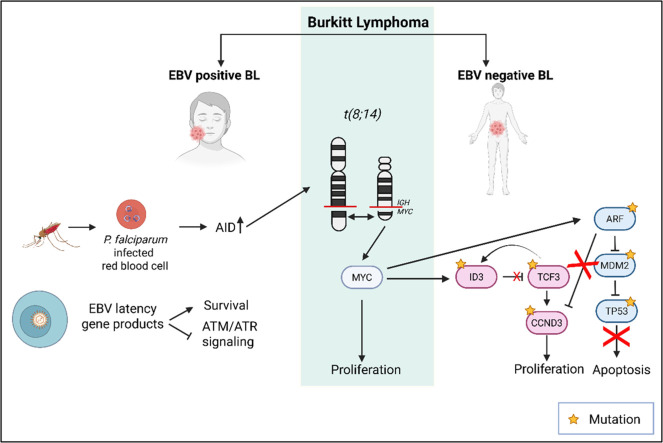

## Introduction

Burkitt lymphoma (BL), named after Denis Parsons Burkitt, is the commonest childhood cancer in sub-Saharan Africa and the most common non-Hodgkin lymphoma (NHL) of children worldwide [[1], [2], [3]]. BL is the fastest growing human tumour; therefore, it is found to be highly sensitive to chemotherapy. BL is potentially curable with multiagent chemotherapy alone [4,5]. Multi-agent chemoimmunotherapy regimens result in long-term progression-free survival for most patients, however, treatment-related mortality in resource poor settings has limited the application of high-intensity regimens [6]. In addition, the long-term side effects of chemotherapy, especially in children, are not negligible (e.g., cardiotoxicity, bone necrosis, limited growth, learning difficulties, infertility, secondary cancers etc.). Despite recent advances in the understanding of molecular drivers of BL, this has not yet been translated into improvements in treatment [6].

BL is traditionally classified into three clinical subtypes based on epidemiological context and geographic location: endemic (eBL), sporadic (sBL), and immunodeficiency-associated BL (Table 1) [[7], [8], [9], [10]]. Endemic BL typically arises in children in Sub-Saharan Africa [4,7,11], where malaria is holoendemic and acquisition of EBV occurs early in life. In non-malaria-endemic regions, BL occurs at a much lower incidence and is termed sporadic BL (sBL). Immunodeficiency-associated BL is linked to HIV infection and may also arise in allograft recipients or those with congenital immunodeficiency [4,7,12].

**Table 1:** Characteristics of the traditional three subtypes of BL [[4], [5], [6], [7],^11^].

Characteristic	Endemic BL	Sporadic BL	Immunodeficiency associated BL
*Geographical distribution*	In Malaria belt, including Equatorial Africa, Papua New Guinea, and Northern Brazil	Worldwide	Worldwide
*Incidence*	5–10 per 100,000 children [12] 74 % of childhood cancers in Equatorial Africa	0.3 per 100,000 people [13] Europe and USA: 30 % of childhood lymphomas and ≤ 3 % of all lymphomas	In HIV infected individuals: ∼20 per 100,000 person [14] In transplant recipients: ∼10 per 100,000 person
*Age*	∼6 years	Children and adults	Mostly above the age of 40 years
*Sex*	Male predominance	Male predominance	Male predominance
*Clinical presentation*	Large facial tumours; jaw and GI tract	GI tract	More aggressive, B symptoms and bone marrow involvement
*EBV positivity*	>90 %	10–30 %	25–40 %
*Other infectious aetiology*	Malaria		HIV

BL is the first human tumour to be associated with a virus, namely, the Epstein-Barr virus (EBV) [5,15]. Dennis Wright, one of the first pathologists to work on BL in Kampala, Uganda, questioned whether EBV-positive BL and EBV-negative BL were different in cell biology and pathogenesis [16]. Today, there is accumulating evidence that EBV-positive and EBV-negative BL are discrete biologic groups based on molecular features regardless of epidemiologic context and geographic location and therefore, supersede the epidemiological subtyping [17]. The 5th edition of the WHO Classification (WHO-HAEM5) has recommended the distinction of EBV-positive and EBV-negative Burkitt lymphoma [17,18].

In this review, we aim to compare and contrast the clinicopathological and molecular features of BL in the context of EBV status. We present the observation that EBV-positive BL may differ by co-infection with malaria; therefore, subgrouping BL based not only on EBV status but also on malaria holo-endemicity may benefit future research and the development of novel therapeutics.

## Epidemiology of BL: malaria, sex, age, HIV and EBV status

### Malaria infection and EBV status

Endemic BL is strongly linked to malaria (*Plasmodium falciparum*) infection. BL is the most common childhood cancer in areas where malaria is holoendemic and acquisition of EBV occurs early in life. This includes equatorial Africa, Papua New Guinea, and parts of Brazil [5,19]. Endemic BL has an incidence of 3–6 per 100,000 children per year, accounting for 74 % of childhood malignancies in the African equatorial belt [12,13]. Despite EBV being a common feature of eBL, not all BL in eBL regions is EBV positive [9]. In practice, cases of eBL are typically not tested for EBV and positivity is assumed. Although intensive chemotherapy with adequate supportive care can typically cure BL, given the majority of eBL occurs in settings with limited resources, it tends to have a poor prognosis [11].

**Fig. 1:**
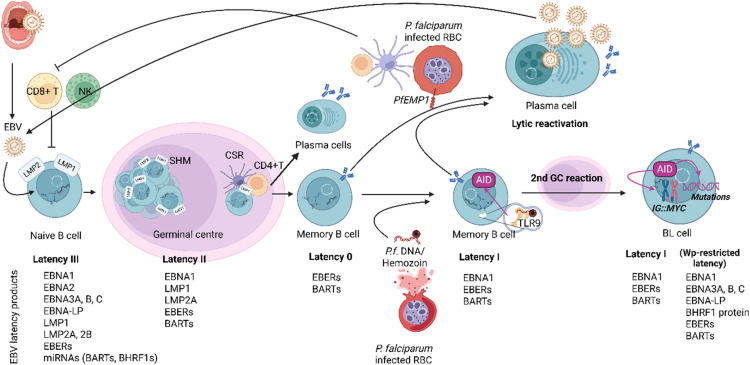
EBV and malaria (*P. falciparum*) co-infection in BL development. EBV primarily spreads through the saliva. Once naïve B cells are infected by EBV, the virus establishes a latent infection in memory B cells. First, EBV infection activates the latency III transcriptional programme, resulting in the expression of nine latent proteins (EBNA1,2,3A,3B,3C,-LP, LMP1,2A,2B), small, non-coding Epstein-Barr virus-encoded RNAs (EBERs), *Bam*HI-A rightward transcript (BART) and *Bam*HI-H rightward open reading frame 1 (BHRF1) miRNAs [23]. Cytotoxic T cells destroy many of these cells, but some survives and enters the GC reaction (Latency II). Then EBV persists in memory B cells without the expression of latent viral genes (Latency 0). Repeated malaria infection induces EBV lytic reactivation through *P. falciparum* erythrocyte membrane protein 1 (PfEMP1), leading to new pool of EBV-infected cells and exhausted anti-EBV T cell immunity [24]. Malaria infection may also induce the clonal expansion of EBNA1 expressing memory B cells (Latency 1) via Toll-like receptor 9 (TLR9) and upregulate the activation-induced cytidine deaminase (AID) [25]. These cells may re-enter into GC reaction. Higher AID causes DNA breaks in the immunoglobulin heavy chain (*IGH*) regions, predisposing to *IG::MYC* translocation; and other mutations (e.g., *TP53*). In about 15 % of eBL, the WP-restricted latency programme is activated which leads to higher resistance to apoptosis [26,27]. (Created in BioRender. Halasz, M. (2025) https://BioRender.com/arjzc9z).

Malaria infection can increase the risk of BL directly through polyclonal stimulation of B cells or indirectly by impairing immunologic control of EBV infection, amplifying the effects of EBV on BL risk (Fig. 1) [20]. Co-infection with malaria plays a crucial role in the development of eBL [21]. EBV infects around 90 % of the global population, yet BL is globally not one of the most common childhood cancers outside areas prone to eBL [22]. Endemic BL is therefore likely to be related to chronic immune stimulation of EBV-containing lymphoid cells by repeated malaria infections rather than EBV latency alone [22]. As will be discussed later, both age of EBV acquisition and co-infection with chronic malaria infection play the most significant role in high incidences of BL in certain regions worldwide.

In non-malaria-endemic regions, BL occurs at a much lower incidence and represents roughly 30 % of paediatric lymphomas but <3 % of all lymphomas of Western Europe and the USA. It has an incidence of 3 cases per 1,000,000 people per year [13]. Sporadic BL has a much lower association with EBV (10–30 %).

### Sex and EBV status

Globally BL has a male predominance. This has been demonstrated in numerous international studies [22,[28], [29], [30], [31], [32], [33]]. Data compiled from the International Incidence of Childhood Cancer, third edition (WHO-IICC-3 website) as part of one study by Muhealdeen et al. demonstrates a clear male predominance of BL worldwide, with the endemic BL of equatorial Africa having less gender disparity than other regions [22].

### Age and EBV status

With eBL, almost all patients are positive for EBV, and the median age at onset of BL is 6 years [6].

The median age of onset of sBL is 30 years [4]. With sBL, only 10–30 % of patients are positive for EBV. SBL has differing age-specific incidence rates depending on geographical location. In Europe, North America, and parts of Asia, sBL is more common in adults, however, in other parts of the world, such as the Middle East, sBL is predominantly a disease of childhood [22]. There is evidence that age of presentation in sBL is related to EBV status. Satou et al. showed that, in Japan, patients with EBV-positive sBL had an older median age (46y vs 13y; *p* < 0.0001) compared to those with EBV-negative sBL, with a higher frequency of patients >50 years of age (48 % vs 14 %, *p* < 0.0001) [15]. In China, childhood rates of BL are low and adult BL is rare. BL in China is rarely EBV positive, yet EBV infects over 90 % of children by ages 5 to 8 [22,34]. A German study of 162 patients with sBL, comprising 92 children/adolescents and 70 adults (>18 years of age), also showed that EBV positivity in sBL was more likely to be detected in adult patients compared to children and adolescents (*p* = 0.0262) [9].

The Middle East is not malaria holo-endemic. The Middle East has a unique pattern of BL, with an incidence of BL lower than equatorial Africa but much higher than any Asian country. Most childhood BL cases are positive for EBV-encoded small RNAs (EBERs) and very few cases of BL are reported in adults. In Egypt and Iraq, EBER has been demonstrated in over 70 % of BL. Age of exposure to EBV is thought to be early childhood in the Middle East, however, there is evidence that a regional change may be taking place, with exposure moving towards adolescence and young adulthood [22]. A study of BL in the Sulaimaniyah Province of Iraq over a 5-year period (2010–2014) showed the median age of BL diagnosis to be 5 years, representing 75 % of all childhood NHL in the region in patients under 10 years of age [35]. The authors speculate that the distinguishing factor epidemiologically between regions with eBL versus BL in the Middle East is early onset chronic malaria infection.

In Brazil, EBV is present in around 50 % of BL [33]. However, Brazil is a diverse country. The North includes the Amazon rainforest and is endemic for malaria, unlike the South. Queiroga et al., analysed 234 cases of BL from five geographic regions of Brazil in paediatric and adult populations. They found the highest frequency of EBV-positive BL was 76 % (13/17) in the North region (where malaria is endemic) and the lowest frequency of EBV-positive BL was 29 % (12/42) in the South region. The North had the youngest mean age (10.3 y) of all regions with 18 % (*n* = 3) of adult and 82 % (*n* = 14) paediatric cases. The South had the oldest mean age (28.6 y) with 62 % (*n* = 26) of adult and 33 % (*n* = 14) paediatric cases (the South region had 2 cases of unknown age) [33]. These demographics partly reflect those of eBL in the North (younger mean age, primarily paediatric population, high EBV positive rates) and sBL in the South (older mean age, primarily adult population, lower EBV positive rates).

The authors believe that the aforementioned geographic trends in BL rates demonstrate the necessity of endemic malaria infection to generate endemic levels of BL among paediatric populations globally and that early EBV infection alone is not enough to account for endemic rates of EBV-positive childhood BL in eBL regions. The findings in the Middle East also support this, as despite most cases of BL occur in childhood and are EBV positive, incidence rates do not reach the endemic levels of equatorial Africa where chronic malaria infection occur at a young age (Table 2).

**Table 2:** Regional patterns of malaria, EBV infection and BL.

Area	Exposure to EBV	Malaria	EBV status of BL	Age of onset (years)	Presentation	Ref.
**Equatorial Africa**	Childhood	Yes	>90 %	6 years	Large facial tumours	[6]
**North Brazil**	Childhood	Yes	76 %	10.3 years	Extranodal > nodal	[33]
**South Brazil**		No	29 %	28.6 years	Extranodal > nodal	
**Iraq**	Childhood	No	20/22 EBV+ (90 %)	5 years	Terminal ileum	[35]
**China**	Childhood	No	88/92 cases EBV+ (95.7 %)	5 years	Extranodal (abdominal commonest)	[34]
**Japan**	Adulthood	No	33/149 cases EBV+ (22 %)	46 years	Tonsil, adrenal glands, Cervical lymph nodes	[15]
		No	117/149 cases EBV-	13 years	GI tract	
**Germany**	Adulthood	No	18/162 cases EBV+ (11 %)	>18 years		[9]
		No	145/162 cases EBV-	<18 years		

### Human immunodeficiency virus (HIV) and EBV status

Despite the use of antiretroviral therapy (ART), lymphoma, including BL, is common in patients with HIV [36]. Immunodeficiency-associated BL was first described in the 1980s [6]. In the setting of HIV, immunodeficiency-associated BL tends to occur early in HIV infection and before CD4+ T-cell counts drop. 40 % of cases are EBV positive [6,13,37]. It is clinically aggressive, and patients tend to have B symptoms and advanced stage disease with bone marrow involvement [4].

Based on observations by one of the co-authors who works as a paediatric oncologist at Muhimbili National Hospital, Dar es Salaam, Tanzania, HIV-positive patients with BL tend to have more extra-nodal disease including abdominal and CNS disease. Head and neck presentations are also different with more lymphadenopathy seen in HIV-positive patients rather than the classic “bony” (mandibular, maxillary or orbital) disease seen in HIV-negative patients.

## Clinical features of BL vary by EBV status and geography

In 2015, Satou et al. compared clinicopathological features of 33 EBV-positive sBL and 117 EBV-negative sBL in Japan [15]. EBV-positive sBL presented more frequently in the tonsils, adrenal glands and cervical lymph nodes, while EBV-negative sBL more frequently involved the GI tract [15]. Huang et al., investigated 92 cases of BL in China (mean age 4.97 years), with 41.3 % cases EBV-positive. The commonest tumour location was the abdomen (95.7 %, *n* = 88/92), regardless of EBV status [31]. In another Chinese study by Bi et al., 43 cases of sBL in children diagnosed between 1990 and 2006, of which only 5 cases (11.6 %) were EBER 1 or 2 positive, were investigated. 36 cases (83.7 %) presented with extranodal lesions and the most common extranodal site involved was abdominal (20/43, 46.5 %) followed by head and neck (14/43, 32.6 %) [38].

Yaqo et al., through analysis of cancer registries, found BL in Middle Eastern countries typically arises in the terminal ileum and contains EBV genomic material in nearly all tumours [35]. Uccini et al. analysed 125 cases of BL among children from Iraq (mean age 5.9 ± 3.1 years), which showed varying clinical presentations, 66 % abdominal and 34 % head and neck. Bone marrow involvement was higher in children with head and neck disease. EBV-encoded RNA was positive in 100/125 (80 %) cases [29]. A single institutional study carried out in a tertiary care university hospital of North India studied 40 cases of BL (median age 11.5 years). 60 % (*n* = 24) patients presented with advanced disease and the primary tumour site was abdominal in 65 % (*n* = 25) cases [30].

A Dutch study by Boerma et al., investigating BL in 107 patients (80 children, 27 adults), showed that children presented with extranodal disease only at a significantly higher rate than adults (48 % v 11 %, *p* < 0.001). Of extranodal sites in children, 34/80 (43 %) had ileocecal involvement with only 7/80 (9 %) having paranasal sinus/jaw involvement [32]. A USA study by Mbulaiteye et al., investigating 296 cases of paediatric BL (mean age of 7.8 years) diagnosed between 1992 and 2005 found that, while 56 % of cases were diagnosed in lymph nodes, the commonest extranodal site for diagnosis was abdominal organs (21 %). Head and facial region accounted for 9 % of diagnostic sites [20].

The commonest extranodal site among sBL appears to be abdominal, regardless of EBV status and geographic location. Regions with high EBV-positive BL rates (Iraq) compared to lower EBV-positive BL rates (China) both had the abdomen as the commonest extranodal site of BL. Studies from India, the Netherlands, and the USA, all demonstrated abdominal presentation as the commonest extranodal site of BL. Although Satou et al. showed contrasting presentations of sBL based on EBV status, with EBV-negative sBL more frequently involving the GI tract, other studies have shown frequent abdominal presentation of sBL in EBV-positive sBL [29,31,35].

The traditionally held view is that in eBL regions, BL typically presents with large tumours of the jaw and/or abdominal cavity, with almost all cells infected with EBV [8,19,37]. A Kenyan study of 961 children and 44 adults with BL showed that, among children, involved sites of the jaw peaked at age 3 and abdomen at age 14, while in adults abdominal presentation was dominant at all ages. In this study 59 % of adult cases showed HIV positivity. HIV BL typically had disseminated disease with lymph nodes, scalp, bone marrow involvement and pancytopenia as major features [39]. Another study investigating clinical characteristics of 471 cases of BL from three regions (Central, Coast, Western) in Kenya showed regional variation in clinical presentation. Age and sex matched cases showed abdominal presentation was commonest in Central Kenya while less common in Coastal and Western Kenya (35 %, 9 %, and 14 %, respectively). Jaw swelling was more common in Western Kenya at 31 %, while Central and Coast were similar at 24 % and 22 %, respectively [40]. A study in Ghana*,* assessing the clinical pattern of eBL in 173 children, all under 13 years of age, from 2007 to 2012, found that the abdomen was the commonest tumour site (46 %) followed by the jaw (31 %) [41]. In Uganda, a study of 1217 cases of childhood BL found the most common presentations to be facial disease (77.65 %) and abdominal disease (69.19 %) [42]. In Northern Uganda, a study of 500 patients with a median age of 6 years found most patients presented with abdominal disease (56 %) versus only facial tumours (35 %). This study used data collected between 1997 and 2006 and based on this information it is suggested that presentation of BL in Northern Uganda has moved from facial to mostly abdominal [43]. Overall, these studies of eBL indicate that along the lymphoma belt of Africa, BL presents most commonly as either or both jaw and abdominal tumours.

In summarising the above information (Table 2, Table 3), the authors note the following observations: Head and neck tumours are more common in endemic compared to sporadic BL regions, however, the characteristic jaw tumour no longer appears to be the dominant presentation of eBL, though it clearly occurs at a higher rate than in sBL regions. The Middle East and parts of Asia are sBL regions but demonstrate higher rates of head and neck lesions compared to other sBL regions. This may be related to the high rates of EBV-positive BL in the Middle East which supports a link between EBV status and head and neck presentation of BL. This association between head and neck presentation and EBV status is further demonstrated by the higher likelihood of head and neck BL in eBL regions. However, abdominal presentation is still more likely than head and neck presentation in both equatorial Africa and the Middle East, in both EBV-positive and negative BL. The dominant extranodal presentation of BL globally appears to be abdominal, regardless of EBV status or incidence rates (endemic vs sporadic).

**Table 3:** Geographical variations in the clinical presentation of BL.

Geographical Location	Dominant clinical presentation	References
Japan	EBV-positive: Tonsils, adrenal glands, cervical lymph nodes	[15]
	EBV-negative: GI tract	
China	EBV-positive: Abdominal	
	EBV-negative: Abdominal, head and neck	
Middle East	EBV-positive: GI tract, head and neck	[29,35]
Northern India	Abdominal	[30]
The Netherlands	GI tract	[32]
The USA	Lymph nodes, abdominal	[20]
Central Kenya	EBV-positive: Abdominal	[39]
Western Kenya	EBV-positive: Jaw swelling	[39]
Ghana	EBV-positive: Abdominal, jaw swelling	[41]
Uganda	EBV-positive: Facial, abdominal	[42]
Northern Uganda	EBV-positive: Abdominal, facial	[43]

## Cell of origin of BL may differ between EBV-positive and EBV-negative BL

It is widely accepted that BL has a germinal centre (GC) origin [[44], [45], [46], [47], [48]]. This is in part due to the immunoglobulin (*IG*) variable genes carrying somatic hypermutation (SHM), a feature characteristic of GC-experienced cells [44,47,49]. In addition, the immunophenotypic, histological, and gene expression features of BL are consistent with a GC B-cell origin [44]. It has also been proposed that the BL cell of origin may differ based on EBV status, with EBV-positive BL reflecting late GC or memory B cells*.* In 2005, Bellan et al. investigated the cell of origin of 31 cases of BL from primary tumours (15 eBL, 10 sBL, 6 AIDS-related BL), of which 18 were EBV positive and 13 were EBV negative. Based on the mutation patterns identified through analysis of somatic mutations and antigen selection, it was suggested that EBV-negative BL reflected early centroblasts that underwent a first round of mutation and reached the subsequent mutation-silent phase prior to antigen selection (low number of somatic mutations, lack of ongoing mutations, no signs of antigen selection (0/13)), while EBV-positive BL reflected late GC B cells that have begun differentiation into memory B cells (higher number of somatic mutations, signs of antigen selection (11/18) in the absence of ongoing mutations) (Fig. 1). The differences were more pronounced when comparing BL by EBV status rather than geographic origin and HIV status [19].

EBV-positive BL has a unique pattern of EBV viral latent protein expression (Fig. 1), restricted to Epstein-Barr virus nuclear antigen 1 (EBNA1), which is required to maintain the viral genome. EBNA1 evades immune detection by cytotoxic T cells. In normal B cells infected with EBV, it was shown that when the cells divide, they express EBNA1 only. This suggests EBV-positive BL may reflect a tumour of latently infected memory B cells stuck proliferating and constitutively expressing only EBNA1 [50]. In keeping with this theory, suppression of EBNA1 in a BL cell line (Raji) significantly inhibited proliferation of the BL cells [51].

## Differences in *IG::MYC* breakpoints in BL based on EBV status

BL is characterized by a recurrent *MYC* translocation that places the *MYC* proto-oncogene next to an immunoglobulin enhancer, leading to constitutive MYC expression [11,12,19,52]. This occurs in BL irrespective of EBV status or geographic location [21].

The *MYC* gene (8q24) encodes the MYC (or c-Myc) transcription factor which regulates cell proliferation, differentiation, and apoptosis [6,13]. MYC regulates up to 15 % of all human genes [53]. The expression of *MYC* is tightly regulated in normal circumstances [54]. *MYC* expression is strictly controlled at both transcription and translation levels, and the half-lives of both the mRNA and protein are very short in normal cells [53,55]. MYC requires positive regulatory signals to promote cell proliferation. In addition, MYC can induce apoptosis under physiological conditions, with MYC overexpression inducing apoptosis by a variety of stimuli [55,56].

In about 75 % of all cancers, MYC is deregulated. In BL, MYC is not activated by oncogenic mutations in the coding sequence, rather cells overexpress the intact MYC protein due to translocation [53]. Alone, the *IG::MYC* translocation is not sufficient to initiate malignant transformation of B cells [8,44,45,54,57]. However, continuous MYC activity is required for BL cell proliferation and survival. Many of the MYC target genes are themselves transcription factors and waves of time-dependent transcription are established through direct and indirect target gene regulation. This creates extensive transcriptional deregulation and oncogene-induced stress, which BL cells are somehow able to tolerate [55].

**Fig. 2:**
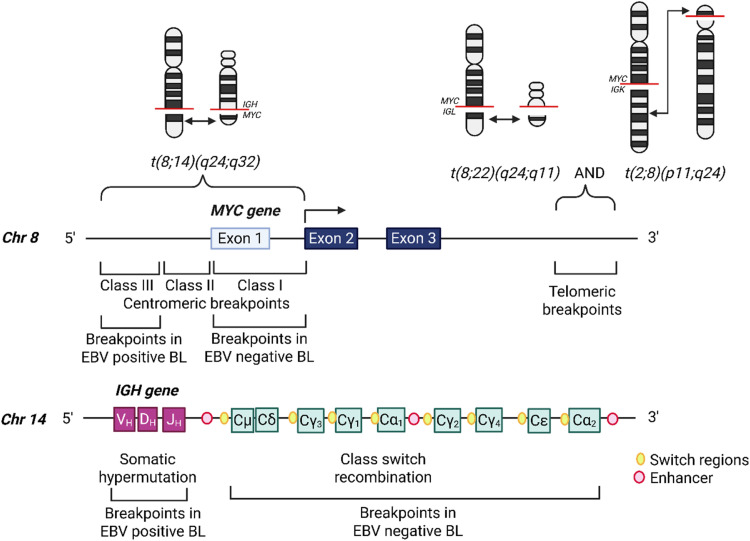
Breakpoints of *IG::MYC* translocations in BL. *MYC* translocations involve the immunoglobulin heavy chain (*IGH*, 14q34), the immunoglobulin lambda (*IGL*, 22,q11.2) or kappa (*IGK*, 2p11.2) light chains. Breakpoints of *IGK::MYC* or *IGL*::*MYC* translocations occur downstream of *MYC* (3′, telomeric). In EBV+ BL, breakpoints of *IGH::MYC* translocations usually occur upstream of *MYC* (class III) and in the *IGHV* regions due to aberrant somatic hypermutation. In EBV- BL, breakpoints of *IGH::MYC* translocations usually occur in the first exon of *MYC* (class I) and in the switch regions of *IGH*, derived from aberrant class switch recombination. (Created in BioRender. Halasz, M. (2025) https://BioRender.com/tj051pc).

*MYC* translocations involve the immunoglobulin heavy chain (*IGH*, 14q34) in 80–85 % of cases, or more infrequently the immunoglobulin light chain kappa (*IGK*, 2p11) or lambda (*IGL*, 22q11) loci (5 % and 15 % of cases, respectively) [4,37]. These translocations have different breakpoints [59,63]. With *t(8;14)*, activation of *MYC* occurs on the derived chromosome 14, with breakpoints upstream (5′, centromeric) of *MYC* or within the *MYC* locus. With *t(2;8)* and *t(8;22)*, the breakpoints are downstream (3′, telomeric) of *MYC* and activation occurs on the derived chromosome 8 in both cases [5] (Fig. 2).

Breakpoints in BL translocations vary depending on EBV status. Breakpoints in EBV-negative BL usually occur in the switch region on the *IG* gene side and in the first exon of *MYC*, which is rationalized by mistakes in normal class switch recombination (CSR) followed by selection of the *MYC* rearrangements capable of promoting cell proliferation [58]. These are class 1 breakpoints, defined as breakpoints within the exon 1 and first intron of *MYC* [53]. In contrast, with breakpoints in EBV-positive BL the *IG* gene has completed VDJ joining and the *IG* gene break point is typically not at the switch junction [58]. The breakpoint in *MYC* is not limited to the first exon, it may occur tens of kb pairs upstream from the first exon [58]. *MYC* breakpoints at the 5′ end of *MYC* located within a few kilobases of exon 1 are termed class 2, and breakpoints distant from *MYC* which can be over 100 kilobases away are termed class 3 [53]. *MYC* activation by EBNA2 is thought to increase the susceptibility of upstream enhancer regions of *MYC* to AID activity, leading to the variation in breakpoints between EBV-positive and negative BL [58]. Through whole-genome sequencing of 212 cases of BL (103 EBV-negative and 109 EBV-positive cases), Thomas et al., characterized the *IGH::MYC* breakpoints in BL and found that EBV-negative BL had significantly more breakpoints attributed to CSR, while EBV-positive BL had significantly more putative SHM-mediated breakpoints. The EBV-positive BL breakpoints upstream of *MYC* could be attributed to aberrant AID activity [59].

## Molecular differences between EBV-positive and EBV-negative BL

The *IG::MYC* translocation is considered a primary oncogenic event in the pathogenesis of BL, however, it is likely that deregulation of other signalling pathways are also required to produce the full malignant phenotype [8,11,52]. Through grouping BL by EBV status, significant differences have been revealed in the mutational landscape of BL.

### ID3/TCF3/CCND3 signalling axis mutations

**Fig. 3:**
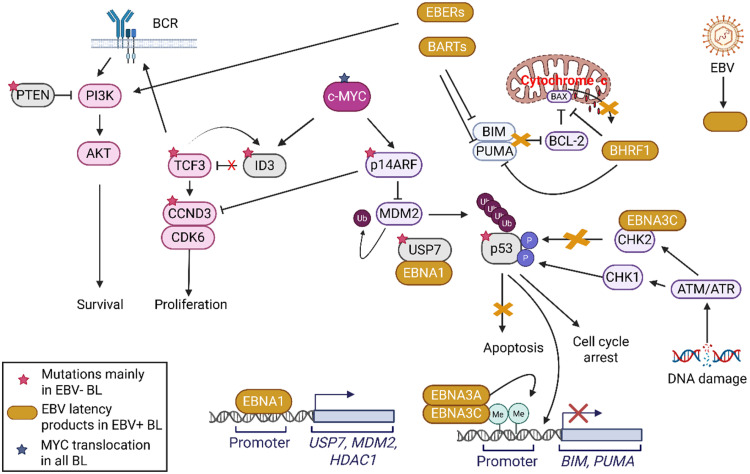
Apoptotic pathway alterations in EBV-positive versus EBV-negative BL. EBV-negative BL is dependent on ID3/TCF3/CCND3 axis deregulation while in EBV-positive BL EBV latency products alter apoptotic signalling. BL is mainly associated with Latency I products (EBNA1, EBERs, BARTs), however, 15 % of endemic BL is characterized by Wp-restricted latency (expressing EBNA-LP, EBNA1, EBNA3A, EBNA3B, EBNA3C, and BHRF1 proteins) [26,27]. EBNA1 may contribute to p53 degradation by upregulating p53-inhibiting genes such as ubiquitin-specific protease 7 (*USP7*), *MDM2*, and *HDAC1* [67]. (Created in BioRender. Halasz, M. (2025) https://BioRender.com/4iytby8).

TCF3 is a master regulator of GC B-cell differentiation [53]. It acts mainly as a transcriptional activator in B cells and regulates the expression of genes critical for GC development. TCF3 is a basic helix-loop-helix (HLH) transcription factor that homodimerizes via its HLH domain and uses its basic region to contact DNA within the major groove of DNA [45]. This DNA binding is inhibited by heterodimerization with ID3, an HLH protein lacking the basic region. In an effort to avoid overexpression, TCF3 activation also leads to transactivation of ID3, which creates a negative autoregulatory loop [45]. TCF3 activity is limited in B cells before activation due to inhibition by ID3 at this time. Upon B cell activation, ID3 is downregulated which leads to increased TCF3 activity [60]. TCF3 promotes survival and proliferation of lymphoid cells via activating the B-cell receptor (BCR)/phosphatidylinositol 3-kinase (PI3K) signalling pathways and modulating cyclin D3 (CCND3) expression (Fig. 3) [61].

ID3-TCF3-CCND3 pathway gene mutations may represent a very relevant second hit in the oncogenesis of BL [57]. *TCF3* and *ID3* mutations in BL relieve TCF3 from the negative influence of ID3 and promote its constitutive activity as a transcription factor [44]. Somatic mutations in *TCF3* can block ID3 binding, while mutations in *ID3* may inactivate it and prevent its effect on TCF3 [53]. Constitutive TCF3 activation ensues and promotes tonic BCR signalling via *PTPN6* repression (PTPN6 encodes a negative regulator of BCR signalling). This tonic BCR signalling induces phosphatidylinositol 3-kinase signalling pathways, which promote BL cell survival [53,55]. TCF3 also activates CCND3 to drive BL cells through cell cycle progression [53]. ID3 mutant proteins are less effective in inhibition of TCF3, thus leading to increased cell proliferation and survival via PI3K and CCND3 [57].

Sander et al. demonstrated that the combination of both constitutive MYC expression and PI3K activity in GC B-cells of a mouse led to BL-like tumours, indicating that survival signals delivered by PI3K counterbalance the pro-apoptotic properties of MYC and together cooperate in BL pathogenesis [62]. In vivo studies performed by Panea et al. investigated the pathogenic contribution of TCF3/ID3/CCND3 dysregulation in the development of Burkitt lymphoma using mouse models. *Id3+/+* or *Id3+/−* mice were crossed onto the *Eu-Myc* background. The *Eu-Myc;Id3+/−* mice developed large abdominal tumours similar to the clinical presentation of sBL and had reduced latency to tumour development compared to *Eu-Myc;Id3+/+* mice (median survival, 70.5 days and 114.0 days, respectively), suggestive that ID3 loss potentiated the effects of MYC in the pathogenesis of BL [7].

There is accumulating evidence that the ID3/TCF3/CCND3 axis is more frequently mutated in EBV-negative BL compared to EBV-positive BL [16]. In the 2016 WHO classification system, ID3 and TCF3 were added to the molecular characteristics of BL [57,61]. Recurrent mutations in *TCF3/ID3* have been identified in sporadic, endemic and immunodeficiency-associated BL, suggesting a common pathogenesis [9]. However, rates of *TCF3/ID3* mutations vary considerably between the clinical subtypes of BL. Next generation sequencing (NGS) studies have shown that mutations in *TCF3* or *ID3* occur in 70 % of sporadic and immunodeficiency-related BL and 40 % of endemic BL, though these rates vary between studies [6,53,57]. Up to 30 % of BL have *CCND3* mutations, which increase intracellular cyclin D3 and contribute to cell cycle deregulation [6].

RNA sequencing of 28 sBL patient samples and 13 BL cell lines by Schmitz et al. found *TCF3/ID3* mutations to be highly recurrent in sBL [45]. Knockdown of *TCF3* caused a time-dependent toxicity in all BL cell lines tested. Introduction of wild type *ID3* into BL cell lines with *ID3* mutations was also lethal. PI3K signalling was shown to be an essential pro-survival mechanism in BL, as all cell lines had PI3K activity and were killed by PI3K pathway inhibitors. PI3K signalling was promoted by constitutive BCR signalling and TCF3. Oncogenic *CCND3* mutations produced stable CCND3 isoforms which drove cell cycle progression in 38 % of cases [45]. Lopez et al. identified 18 putative driver genes through whole genome and transcriptome sequencing of 39 cases of EBV-negative sBL. This included *ID3, TCF3, CCND3* and *TP53*. 74 % (29/39) of cases had an *ID3* and/or *TCF3* alteration identified [63]. Richter et al. sequenced 91 cases of sBL from central Europe (67 adult and 24 paediatric cases) and identified 299 different single nucleotide variants and small insertions or deletions (indels) affecting 22 genes or “hotspot” regions among 88/91 cases. In the remaining 3 cases, no variant was detected. Among these 88 cases, 75 (86 %) had a mutation in at least one gene of the ID3/TCF3/CCND3 pathway. Of the remaining 13 cases, 11 had variants affecting other genes known to be mutated in BL, such as *GNA13, FOXO1, THOA, MYC, ARID1A*, and *TP53*. Mutations affecting *ID3/TCF3/CCND3* were significantly more likely in EBV-negative sBL, irrespective of age (*p* = 0.0088), while mutations of G protein subunit alpha 13 (*GNA13; p* = 0.0368) and forkhead box O1 (*FOXO1; p* = 0.0044) were significantly more likely in EBV-positive sBL [9]. Abate et al. performed RNA-sequencing on 20 cases of eBL and compared the mutational landscape of eBL with published data on sBL [10]. The authors demonstrated lower mutational frequencies of *ID3, TCF3, CCND3, MYC, DDX3X* and *TP53* in eBL samples compared to sBL, and higher mutational frequencies in *ARID1A, RHOA*, and *CCNF*. In cases of sBL there was almost mutual exclusivity between EBV positivity and *TCF3/ID3* mutations (*P* < 0.02, Fischer exact test). The TCF3 pathway was clearly more activated in EBV-negative cases, indicated by significant negative enrichment of TCF3 target genes in EBV-positive samples. The mutual exclusivity of *TCF3/ID3* mutations and EBV status was more significant than clinical subtype (*p* < 0.0008, Fisher exact test) [10].

Overall, these results suggest that EBV-negative BL is far more dependent on ID3/TCF3/CCND3 axis deregulation in both the development and survival of BL cells compared to EBV-positive BL. As a result, this pathway may represent a therapeutic vulnerability for EBV-negative BL. Targeting the PI3K pathway, tonic BCR signalling, and cyclin D3/CDK6 has been previously proposed in BL [45].

### Evading apoptosis

Overexpression of MYC has been shown to induce apoptosis in normal B cells [5]. Deregulated expression of MYC in normal B cells stimulates the accumulation and activation of p53. This is achieved mainly through MYC stimulation of expression of p14ARF from the *CDKN2A* gene [21]. P14ARF antagonizes MDM2, the negative regulator of p53. As a result, p53 accumulates and drives apoptosis [21,64,65].

The presence of apoptotic gene mutations in BL can differ significantly between EBV-positive and negative BL (Fig. 3). Grande et al. reviewed potential driver mutations in BL by analysis of structural and copy number variations affecting BL-associated genes. They identified significantly more frequent *TP53* mutations (*Q* = 0.0044, Fisher’s exact test) in EBV-negative BL (but not sBL), which became more striking when considering all BL-associated genes with roles in apoptosis (*Q* = 0.00024) [11]. When comparing mutational frequencies in BL by EBV status, Thomas et al. identified 13 differentially mutated genes (*FOXO1, MIR17HG, PTEN, SMARCA4, GNAI1, CCND3, TP53, CDKN2A, SYNCRIP, FBXO11, STAT6, BCR, PHF6*). All apart from *FOXO1* and *BCR* were mutated at a higher frequency in EBV-negative BL. When the significantly mutated genes were assigned to oncogenic pathways, genes related to apoptosis were found to be more commonly mutated in EBV-negative BL [66]*.* Abate et al. also demonstrated lower mutational frequencies of *TP53* were observed in eBL compared to sBL [10]. All 40 cases of eBL were EBV positive while 4/27 (15 %) cases of sBL were EBV positive, with the authors describe “a dual mechanism of transformation (mutation versus virus driven in sBL and eBL, respectively)” [10].

Thomas et al. performed whole-genome sequencing of BL (*N* = 230) and diffuse large B-cell lymphomas; and reported three subgroups of BL: DGG-BL (mutations in *DDX3X, GNA13*, and *GNAI2*), IC-BL (mutations in *ID3* and *CCND3*), and Q53-BL (quiet *TP53*) [68]. In this study, the Q53-BL subgroup enriched for *TP53* mutations was associated with EBV negativity.

#### ARF/MDM2/p53 pathway

The p53 pathway acts as a protective mechanism against MYC-induced lymphomagenesis. The p53 protein levels are normally kept low by its negative regulator, MDM2 which induces its proteasomal degradation [65]. P53 is present at low levels in resting and is activated by a series of post-translational modifications that free it from MDM2 [69]. P53 activation requires its stabilisation via MYC/FOXO transcription of the *CDKN2A* locus encoding p14ARF, which inhibits MDM2 induced degradation of p53 [55]. The apoptotic mechanisms of p53 are well documented and includes regulation of transcription of BCL family members such as BAX, BAK, and PUMA, as well as regulation of BCL family member function via direct protein-protein interactions [64].

Inactivating mutations and deletions affecting the ARF/MDM2/TP53 axis are commonly found in BL cell lines [65,70]. Eischen et al. investigated transgenic mice expressing the *Myc* oncogene driven by *IgH (Eμ)* enhancer and the development of B-cell lymphoma. In cultured primary mouse embryo fibroblasts, MYC activated the ARF-MDM2-p53 tumour suppressor pathway, enhancing p53-dependent apoptosis. Ultimately cells that had either a *Tp53* mutation or biallelic *Cdkn2a* deletion survived. The authors concluded that *Myc* activation strongly selected for spontaneous inactivation of the ARF-MDM2-p53 pathway in vivo, cancelling its protective checkpoint function and accelerating progression to malignancy [71]. Lindström et al. found the ARF-MDM2-p53 pathway is frequently inactivated in BL cell lines [72]. They found that 19 primary BL biopsies had retained an intact *CDKN2A* gene and expressed p14ARF. Of the 47 established BL cell lines, 3 (6 %) had p14ARF loss by homozygous deletion of *CDKN2A*. When Lindström et al. assessed the 7 BL cell lines with wild type *TP53*, 3/3 BL lines with homozygous *CDKN2A* deletion were EBV negative, while all 4 with retained *CDKN2A* were EBV positive [72]. This points to EBV-related differences in *CDKN2A* status of BL, although as the authors also note, the sample size is “too small to draw firm conclusions” [72].

#### Anti-apoptotic effects of EBV latency gene products

Many of EBV’s latency genes target apoptotic pathways, leading to inhibition of apoptosis in EBV-positive BL cells (Fig. 3) [73]. Evidence of EBNA1 having anti-apoptotic activity is now compelling [21]. The significantly fewer mutations in genes affecting apoptosis observed in EBV-positive BL compared to EBV-negative BL support the theory that EBV plays a direct role in inhibition of apoptosis in BL. This theory is supported by studies that found when EBV is removed from EBV-positive cell lines, they die by apoptosis [73].

Latency-associated EBV gene products can inhibit a variety of apoptotic and senescence promoting pathways, thus working against the proliferation-restricting activities of deregulated MYC and playing an important role in the pathogenesis of EBV-positive BL. At least 9 of the EBV latency-associated gene products (EBNA1, EBNA2, EBNA3A, EBNA3C, LMP-1, LMP-2A, miR-BART5, EBERs, BHRF1) have been linked to enhanced cell survival, capable of resetting the apoptosis threshold and thus may contribute to the pathogenesis of EBV-positive BL (reviewed in [21]). A subset of eBL has been associated with the Wp-restricted latency programme (Fig. 1), expressing EBNA1, EBNA3A, EBNA3B, EBNA3C, EBNA-LP and BHRF1 proteins [26,27].

Although EBV-positive BL tumours typically do not express so many gene products (latency I, see Fig. 1), the “hit-and-run” theory should be considered. As per this theory, it is postulated the virus is essential for initiating oncogenesis and the viral genome is later lost in an effort to evade immune surveillance while host mutations accumulate in the cell [74]. The “hit” involves EBV providing a normal B cell with potential oncogenic growth and survival information. The “run” occurs as EBV avoids elimination of infected cells by inducing epigenetic alterations and silencing targeted viral genes in the host cell [23,74]. This theory has even been suggested for EBV-negative BL. A study investigating 10 cases of BL diagnosed as EBV negative by IHC and EBER-ISH demonstrated EBV-microRNAs and EBV genome when assessed by applying conventional methods of EBV detection alongside non-conventional methods including EBV-microRNAs detection and EBV viral load measurement. They found that the 6 cases diagnosed as EBV negative by IHC and EBER-ISH harboured EBV-microRNAs and EBV genome [74]. Another study, in which a clonal CCL185 human lung carcinoma cell line was infected with recombinant EBV, demonstrated that EBV infection led to epigenetic alterations in host cells that were maintained even after loss of the virus [75].

#### EBV and the ATM/ATR pathway

Another pathway of MYC-induced activation of p53 is via the Ataxia-Telangiectasia mutated (ATM) / ATM and RAD3-related (ATR) kinase pathway. These kinases are master regulators of the DNA-damage response network, activated by double-stranded DNA (dsDNA) breaks. Both kinases can phosphorylate and stabilise p53 by inhibiting its interaction with MDM2. Studies have demonstrated that loss of these kinases impairs MYC-induced apoptosis and enhances cell proliferation [65].

EBV infection disrupts ATM-mediated DNA damage response via downregulation of ATM expression and inhibition of its downstream effectors. ATR has been identified as a key molecular switch that EBV targets to overcome checkpoint arrest during transformation [76].

Choudhuri et al. found that EBNA3C can disrupt the G2/M cell cycle checkpoint arrest induced by the drug nocodazole and allow cell cycle progression. The direct interaction of EBNA3C with CHK2, the ATM/ATR signalling effector, was responsible for the release of this nocodazole-induced G2/M arrest [77]. In addition, Krauer et al. demonstrated that the ATM/ATR signalling in G2/M checkpoint responses to drugs azelaic bishydroxamine, etoposide (Etoposide), and hydroxyurea may be blocked by the EBNA3 family proteins [78].

#### EBV and deregulation of BCL-2 family proteins

The BCL-2 homology 3 (BH3) only protein PUMA (TP53 upregulated modulator of apoptosis) indirectly activates pro-apoptotic proteins BAX and BAK, which initiate the intrinsic apoptotic pathway via disruption of the outer mitochondrial membrane leading to mitochondrial cytochrome c release [55,65]. MYC-mediated repression of anti-apoptotic BCL2 members has been demonstrated in vitro and in vivo and sensitises cells to apoptotic stimuli [65]. BIM (BCL-2-initiating mediator of cell death) directly initiates apoptosis by binding with high affinity to BCL-2 and the other pro-survival family members to inactivate them [21]. BIM also binds and activates BAX to initiate cytochrome c release from mitochondria [21]. It has been shown through expression profiling and functional analysis of apoptosis-related proteins and transcripts in BL cells that EBV inhibits upregulation of BIM and PUMA, thus enhancing the survival of BL cells by suppression of the intrinsic apoptotic pathway [79].

A study by Richter-Larrea et al. demonstrated that *BIM* epigenetic silencing frequently occurs in both primary BL samples and in BL cell lines [80]. EBNA3A and EBNA3C functionally interact to inhibit *BIM* expression in EBV-positive BL via epigenetic mechanisms. *BIM* is an important tumour suppressor gene in B cells and its inhibition by EBNA3A and EBNA3C likely plays a major role in EBV-associated B cell lymphomagenesis. EBNA3A and EBNA3C are not generally expressed in EBV-positive BL, however their inhibition of *BIM* is epigenetic and thus heritable, making their continued expression unnecessary in EBV-positive BL [21]. This supports the “hit-and-run” theory of EBV.

In BL with Wp-restricted latency, BHRF1 is expressed which binds to the BH3 domain of pro-apoptotic BIM, BID, BAK and PUMA, acting as the viral homologue of the pro-survival protein BCL-2 [81].

From the above referenced studies, EBV-negative BL therefore relies on oncogenic mutations in apoptotic pathways, especially the ARF-MDM2-p53 pathway, while EBV-positive BL gains anti-apoptotic activity as a direct effect of EBV on apoptotic pathways involving (but not limited to) ATM/ATR, BIM, and PUMA (Fig. 3). Therefore, the ARF-MDM2-p53 pathway may represent a therapeutic vulnerability for EBV-negative BL, while targeting the EBV latency products may be the Achilles’ heel for EBV-positive BL.

### Epigenetic modifications

Aberrant methylation of CpG islands is frequently detected in lymphomas. This modification is catalysed by DNA methyltransferases (DNMTs). Overexpression of DNMT1 and DNMT3B has been observed in Burkitt lymphoma (BL) tumour samples, independent of Epstein–Barr virus (EBV) status [82].

EBV can induce methylation of tumour suppressor genes. A 2025 study looking at the DNA methylome in BL found that EBV-positive BL displayed significant hypermethylation patterns in the DNA methylome compared to both EBV-negative BL and normal centroblasts [83].

A separate study profiled 96 cases of BL, 17 BL cell lines and 6 EBV-transformed lymphoblastoid cell lines, with DNA methylation analysis clustering cases into 4 subgroups: 2 containing mostly EBV-positive and 2 containing mostly EBV-negative cases. They found that subgroups containing mostly EBV-positive cases showed increased DNA methylation compared to subgroups containing mostly EBV-negative cases. EBV-associated hypermethylation affected regulatory regions of genes frequently mutated in BL (e.g., *CCND3, TP53*) and impacted super-enhancers, suggesting that hypermethylation may compensate for the lower mutational burden of pathogenic drivers in EBV-positive BL [84].

Therefore, therapeutic targeting of epigenetic modifications (e.g., DNA methyltransferase (DNMT) inhibitors, histone deacetylase (HDAC) inhibitors, etc.) are a promising avenue for BL [85]. For instance, Robaina et al. showed that the DNMT inhibitor decitabine (Dacogen) reduces the expression of DNMT1 and DNMT3B; and inhibits cell growth in the BL41 and Raji BL cell lines [82]. Mieland et al. reported that the HDAC10 inhibitor PZ48 disrupts the MYC-DNA polymerase POLD1 regulatory loop (in which MYC stabilizes POLD1, which in turn increases MYC expression and proliferation), thereby inducing DNA damage and apoptosis in the Ramos BL cell line and in ALL cell lines [86]. Li et al. showed that the HDAC inhibitor valproic acid (Depakene) leads to increased acetylation of the *PTEN* promoter which in turn inhibits PI3K/AKT signalling and proliferation in Raji and CA46 BL cell lines [87].

### HIV and BL

The mutations found in HIV-related BL include *IG::MYC* translocation, frequent inactivation of *TP53*, and point mutations in *BCL6* [88]. HIV may drive lymphomagenesis directly. The HIV-1 matrix protein p17 persists in the germinal centres and may upregulate the EBV oncoprotein LMP-1 (latent membrane protein) in EBV-infected B cells. It might also promote the growth of transformed B cells via the AKT pathway. The trans-activator of transcription HIV protein causes increased expression of the activation-induced cytidine deaminase (AID), an enzyme required for class switch recombination from IgM to other immunoglobulin subclasses in the germinal centre [89].

### Co-infection of chronic malaria with early EBV infection leads to aberrant AID expression

Co-infection of chronic malaria with early EBV infection leads to aberrant AID expression and this co-infection is the most likely cause of endemic BL. AID is an enzyme essential for the processes of SHM and CSR of antibody genes in B cells during an immune response, leading to a diverse and more efficacious antibody response [90]. AID deaminates cytosine in DNA, converting it to uracil and leading to a U:G mismatch lesion. These lesions are converted to point mutations during SHM and into double-strand DNA breaks during CSR [91]. Properly functioning, AID activity is primarily restricted to *IG* genes. Off-target activity for non-*IG* loci can lead to chromosomal translocations, and *MYC* has been identified as a target of AID [73]. The induction of double-strand DNA breaks by AID in genes apart from *IG* genes by aberrant AID expression has been linked to the development of malignancy [92]. Mistargeted AID activity is a major cause of oncogenic translocations [91]. In most cases of BL it is generally accepted that chromosomal translocations are mediated by aberrant SHM and CSR, requiring AID activity. Activated AID can directly mediate translocation of *MYC* in GC B cells [21].

Integrative genomic and transcriptomic characterization of 106 paediatric BL tumours (all HIV negative) was performed by Grande et al., to compare EBV-positive BL (*n* = 71) with EBV-negative BL (*n* = 35) [11]. EBV-positive BL demonstrated a higher mutational load with higher rates of AID expression and subsequent aberrant SHM [11]. In 2019, Panea et al. performed WGS and transcriptome sequencing to 101 tumour-normal pairs (60 sBL, 32 eBL and 9 immunodeficiency-associated BL) [7]. RNA was available for 82/101 cases. 81 % of eBL were EBV positive, compared with 20 % of sBL. EBV-positive BL had a significantly higher somatic mutation load than EBV-negative BL, with a higher proportion of AID-associated mutations present [7].

Globally, EBV-positive BL occurs at considerably lower rates in malaria non-holoendemic regions compared to malaria holoendemic regions. Chronic malaria infection clearly augments the risk of BL when present alongside EBV. AID activity may play a key role in explaining how malaria impacts on BL development [93]. Wilmore et al. investigated AID expression in peripheral blood mononuclear cells of children in Western Kenya with endemic and sporadic malaria transmission dynamics. In children from the malaria endemic region, detectible EBV viral load was associated with higher AID expression than children with undetectable EBV. This difference was not seen in children with sporadic exposure to malaria [93]. In fact, AID levels in EBV-positive children from the sporadic malaria region were comparable to EBV-negative children [93]. This suggests high frequency of malaria infection in the presence of EBV, which would only occur in malaria endemic regions, correlates with increased AID expression. In the case of BL, this would indicate that AID activity is particularily important to the pathogenesis of EBV-positive BL in malaria holoendemic regions.

Robbiani et al. used *Plasmodium chabaudi* (*P. chabaudi)* to produce chronic malaria infection in mice, and found the infection led to prolonged expansion of GCs in which B cells express AID. The GC B cells elicited during the *P. chabaudi* infection had widespread DNA damage leading to chromosomal translocations. *P. chabaudi* infection led to lymphomagenesis that favoured mature B cell lymphoma that are AID-dependent and harbour chromosomal translocations [94]. Kalchschmidt et al. demonstrated that the EBV oncoprotein EBNA3C induces expression of AID in EBV-infected cells. CD19+ B cells were infected with previously constructed and characterised EBNA3C KO and revertant EBV recombinants. Infection with EBNA3C revertant virus led to gradual induction of AID mRNA to high levels over 30 days, while this induction of AID was not seen after infection with EBNA3C KO virus. Rather, the later led to AID mRNA levels similar to those seen in uninfected primary B cells [95]. Torgbor et al. showed, through in vitro analysis of tonsil B cells incubated with malaria extract prepared by lysing RBCs infected with *P. falciparum,* that *P. falciparum* would stimulate AID expression in the GC, concluding that *P. falciparum* is a potent antigen-independent stimulator of AID expression. The authors also performed in vivo analysis of purified GC cells from tonsils of patients with and without malaria infection. The levels of AID mRNA were significantly higher in tonsils of patients with malaria compared to the controls [96]. EBV- and *P. falciparum*-directed immune responses trigger the expression and activity of AID, which has oncogenic consequences [97]. In experimental settings, AID can induce *IG::MYC* translocations similar to those found in BL [44,97]. Aberrant AID activity has been shown to mediate DNA damage that produces dsDNA breaks in *MYC* that lead to *IGH::MYC* translocations in primary B lymphocytes [98].

Aberrant AID expression in cases of early EBV infection and chronic malaria co-infection may explain why EBV-positive BL occurs at considerably higher rates in malaria holoendemic regions compared to malaria non-holoendemic regions. Aberrant AID expression may promote the higher rates of BL via increasing the risk of its characteristic translocation. Co-infection with EBV and chronic malaria infection alone does not guarantee the development of BL given the majority of children in these regions do not develop BL, despite the likelihood of children being infected with EBV and chronic malaria at an early age [99]. However, the risk significantly increases as demonstrated by incidence rates compared to the rest of the world. This provides a plausible molecular explanation to why co-infection with early EBV and chronic malaria causes endemic levels of BL. Further evidence that supports the role of malaria infection in the high incidence of BL in equatorial Africa can be seen by assessing the impact of mosquito bed net use on BL rates in equatorial Africa. A systematic review and meta-analysis by Schmit et al. found that a large-scale introduction of insecticide-treated bed nets (ITN) between 1990 and 2017 in sub-Saharan Africa was associated with a reduction in BL among children [100]. In Tanzania, a reduction in malaria transmission has led to considerable reductions in rates of BL [101]. Clinical observations from one of the co-authors of this paper are in line with this: It has been noted that due to an almost successful attempt to completely eradicate malaria from Zanzibar, clinicians rarely ever see BL from the island. In addition, in the Kilimanjaro region, which is higher than the rest of Tanzania and thus has far less, if any, mosquito presence, there are hardly ever cases of BL seen in this region. The lake zone of Tanzania is the most suitable environment for mosquitos to thrive and consequently sees the highest rates of BL. However, even in this area a reduction in BL has been seen, likely attributable to malaria reduction efforts.

From the above studies, we make the observation that early EBV infection combined with chronic malaria co-infection is the defining environmental/acquired feature of EBV-positive endemic BL compared to EBV-positive sporadic BL. Early age of EBV infection (even if at endemic levels during childhood) outside malaria holo-endemic regions cannot alone deliver endemic levels of BL. Evidence for this is seen when considering BL in China and the Middle East, which has been discussed earlier. The oncogenic impact of EBV may require environmental triggers to contribute the oncogenic potential.

*P. falciparum* is highly prevalent in South-East Asia, Eastern Mediterranean, and Western Pacific regions. The most vulnerable groups in high-transmission areas are children younger than 5 years old. Climate change is predicted to lead to an increase in the geographical distribution of malaria and a more suitable climate for malaria transmission in tropical regions [102]. Therefore, the authors believe that it is conceivable that with climate change the global landscape of BL may also change.

The need of EBV to co-operate with an environmental trigger in the delivery of endemic levels of a malignancy is also seen in EBV and nasopharyngeal carcinoma. In Southern China, EBV infection is said to occur at an early age. Environmental agents acting in conjunction with the host’s genetic background are thought to impair immune control of EBV infection, eventually leading to nasopharyngeal carcinoma, which occurs in Southern China at endemic levels [103]*.* Based on this, the authors suggest that if EBV alone was to account for endemic levels of nasopharyngeal carcinoma, it would be endemic worldwide, as would BL.

## Therapeutic considerations

BL patients are typically treated with intensified combination chemotherapy in conjunction with the anti-CD20 monoclonal antibody rituximab [[104], [105], [106]]. Although these regimens have improved survival rates, major challenges remain in mitigating treatment-related toxicity and managing refractory or relapsed disease. Advances in molecular profiling of BL have substantially deepened our understanding of the distinct molecular mechanisms underlying EBV-positive and EBV-negative BL. Translating these insights into less toxic targeted therapeutic approaches is essential for improving clinical outcomes and long-term quality of life for patients with BL.

Advances in the understanding of EBV latency programs, viral antigen expression, and host-virus interactions have opened new avenues for the development of targeted interventions for EBV-positive BL. Emerging strategies focusing on exploiting EBV-associated molecular vulnerabilities include targeting the latency program (e.g., EBNA1 inhibitor), viral lytic induction, targeting AID, and the use of epigenetic modifiers (e.g., HDAC inhibitors) [[107], [108], [109], [110]]. In addition, prophylactic EBV vaccines, immune checkpoint inhibitors, and adoptive cell therapies have also been proposed for EBV-positive BL [111,112].

A recent review by Atallah-Yunes et al. provides a comprehensive overview of small molecule inhibitors under investigation for BL. These compounds include inhibitors targeting the BCR/PI3K/AKT/mTOR pathway, agents that suppress *MYC* transcription or disrupt MYC function, as well as molecules that interfere with tumour cell metabolism or alter cell cycle and apoptosis regulation through inhibition of CDK4/6 or modulation of anti-apoptotic proteins such as MCL-1 [111,113]. Together, these developments highlight a growing emphasis on more precise and less toxic therapeutic approaches for both EBV-positive and EBV-negative forms of BL.

## Future perspectives and conclusions

Based on our review of clinical and molecular variations in BL, we conclude that EBV-positive BL and EBV-negative BL harbour significant molecular differences, supporting their classification as distinct biological entities (Table 4). The most prominent differences identified to date include variations in cell of origin, *IG::MYC* translocation breakpoints, mutation rates in the ID3/TCF3/ CCND3 pathway, aberrant AID expression, SHM patterns, and mechanisms of apoptosis evasion.

**Table 4:** Characteristics of EBV-positive and EBV-negative Burkitt lymphoma.

Characteristic	EBV-positive BL	EBV-negative BL
*Age*	Younger median age	Older median age but regional variation is significant
*Sex*	More common in males	
*Geographical distribution*	Mostly in Malaria belt	Worldwide
*Clinical presentation*	Head and neck; GI tract	GI tract
*Cell of origin*	Late germinal centre (GC) or memory B cells	Early centroblasts
*IgH::MYC breakpoint region*	Class 2,3 breakpoints (Upstream of *MYC*)	Class 1 breakpoints (Within 1st exon/intron of *MYC*)
*Mechanism of t(8;14)*	Somatic hypermutation (SHM), higher AID expression	Class-switch recombination (breakpoints within switch sequences)
*Molecular alterations*	Antiapoptotic effects of EBV latency gene products	*TCF3/ID3/CCND3* pathway mutations
	ATM/ATR kinase pathway alterations	*TP53* mutations
	Downregulation of BIM and PUMA	*CDKN2A* homozygous deletions

Future research focusing on the molecular distinctions of EBV-positive BL arising in malaria holo-endemic versus non-holo-endemic regions could reveal variations in the disease’s molecular landscape. Such work has the potential to clarify whether malaria enhances the oncogenic effects of EBV or contributes its own independent oncogenic alterations driving BL pathogenesis. Ultimately, these insights could lead to new therapeutic targets/benefits for regions of the world suffering most from BL.

Given the disproportionately poor outcomes in resource-limited settings, where the overwhelming majority of BL cases are EBV positive, continued investigation into the combined role of EBV and malaria in BL pathogenesis is essential. This research may uncover novel molecular pathways and therapeutic targets, offering hope for improved treatment and survival for patients from the regions most affected by BL.

## Limitations

In our review of the literature, studies specifically investigating clinicopathological and molecular differences in Burkitt lymphoma based on translocation subtype (i.e., *t(8;14)(q24;q32)* versus *t(2;8)(p12;q24)* or *t(8;22)(q24;q11)*) were scarce. Although each of these translocations ultimately results in dysregulated overexpression of the MYC oncogene, subtle clinical or biological distinctions among them may exist and are not comprehensively addressed here. Accordingly, our discussion primarily centres on BL harbouring the *t(8;14)* translocation, which represents most *IG::MYC* rearrangements in BL and is the most extensively characterized in the literature.

Furthermore, this review focuses on the most prominent and widely discussed molecular variations in BL as reported to date. It is possible that emerging or less widely published findings were not captured within this paper. Our aim was to provide a comprehensive overview of the key molecular differences currently recognized in BL, with particular emphasis on factors associated with EBV infection and malaria co-infection, which remain central to understanding the biological heterogeneity of this disease.

## Funding

This research was supported by The Pathological Society of Great Britain and Ireland (TSGS 0425 03), and the Children’s Health Foundation under the Research Ireland Frontiers for the Future Programme (21/FFP-P/10130).

## CRediT authorship contribution statement

**Eoghan O’Connor:** Writing – original draft, Visualization. **Patricia Scanlan:** Writing – review & editing. **Owen Patrick Smith:** Writing – review & editing. **Melinda Halasz:** Writing – review & editing, Visualization, Supervision, Conceptualization.

## Declaration of competing interest

The authors declare that they have no known competing financial interests or personal relationships that could have appeared to influence the work reported in this paper.
